# Research Progress on Hepatitis E Virus Culture

**DOI:** 10.3390/pathogens14050456

**Published:** 2025-05-06

**Authors:** Jie Zhang, Ziteng Liang, Fan Liu, Youchun Wang, Weijin Huang, Jianhui Nie

**Affiliations:** 1 Division of HIV/AIDS and Sexually-Transmitted Virus Vaccines, Institute for Biological Product Control, National Institutes for Food and Drug Control (NIFDC), Beijing 102629, China; zhangjie2024@nifdc.org.cn (J.Z.); liangziteng@nifdc.org.cn (Z.L.); liufanfan@cau.edu.cn (F.L.); 2 State Key Laboratory of Drug Regulatory Science, National Institutes for Food and Drug Control (NIFDC), Beijing 102629, China; 3 National Institutes for Food and Drug Control, Chinese Academy of Medical Science & Peking Union Medical College, No. 9 Dongdan Santiao, Dongcheng District, Beijing 100730, China; 4 Institute of Medical Biology, Chinese Academy of Medical Science & Peking Union Medical College, Kunming 650118, China; wangyc@nifdc.org.cn

**Keywords:** Hepatitis E virus, cell culture, infectious clones, organoids

## Abstract

Hepatitis E virus (HEV) is a zoonotic pathogen and the main cause of acute viral hepatitis in China, resulting in a significant burden on public health. Developing a highly efficient in vitro culture system for HEV is crucial for understanding the determinants of HEV infection in humans and other animals, the pathogenic mechanisms, as well as the screening and evaluation of antiviral drugs. In this paper, the research progress on HEV in vitro culture systems is reviewed to provide a convenient reference for further research on HEV, aiding comprehensive efforts toward the widespread prevention and control of related diseases.

## 1. Introduction

Hepatitis E (HE) is a zoonotic disease caused by HEV [1]. From a broader perspective, HEV is also one of the major pathogens threatening global public health [2,3]. HEV is classified into the *Hepeviridae* family, which included two subfamilies until 2022: *Orthohepevirinae* and *Parahepevirinae*. The *Orthohepevirinae* includes four genera: *Paslahepevirus* (referred to as HEV-A), *Avihepevirus* (referred to as HEV-B), *Rocahepevirus* (referred to as HEV-C), and *Chirohepevirus* (referred to as HEV-D) [4]. The genus *Paslahepevirus* includes two species, *P. alci* and *P. balayani*. The *P. balayani* species can be divided into eight virus genotypes, namely HEV-1 to HEV-8 [5]. It is estimated that more than 20 million people are infected with HEV worldwide every year, 3.4 million of which develop symptoms of HE, resulting in 70,000 deaths and 3000 stillbirths. The recombinant Hepatitis E vaccine (Hecolin) is now licensed in China and represents a significant advancement in preventing HEV infection [6]. However, the therapeutic arsenal is limited to ribavirin and pegylated interferon-alpha (PegIFN alpha) [7,8,9]. HEV usually causes acute infection only in healthy individuals and the infection is self-limited. However, HEV can cause chronic infection in immunocompromised people, such as organ transplant recipients, patients with human immunodeficiency virus (HIV) infection, and patients with hematological malignancies [10]. Studies have confirmed that HEV can be excreted through urine [11], so in addition to the traditionally recognized fecal–oral route and blood route, there is also a urine–oral route of transmission, increasing the risk of disease spread. As HEV has been isolated from a variety of animals such as pigs [12], wild boars [13], rabbits [14], camels [15,16,17], ferrets [18], deer [19], etc., it has become clear that its zoonotic nature also increases the pressure on prevention and control efforts. The establishment of an efficient in vitro cell culture system for HEV is crucial for the evaluation of the determinants of HEV infection [20], pathogenic mechanisms [21], and antiviral drug screening [22,23], while also being more in line with animal welfare goals than the use of animal infection models. In this review, we summarize the research status of HEV in vitro culture systems, aiming to provide a convenient reference for further research on HEV, which will greatly aid the comprehensive prevention and control of related diseases.

## 2. Virological Characteristics of HEV

### 2.1. HEV Virion Morphology

It is worth noting that, similar to HEV, HEV has been confirmed to occur in two forms: non-enveloped virus particles mainly present in bile and feces and quasi-enveloped virus particles mainly present in blood or tissue culture, with the diameters of the virus particles being 26.9 ± 0.9 nm and 39.6 ± 1.0 nm, respectively [24]. It is speculated that the absence of an envelope improves the infection efficiency by exposing the capsid protein, while the membrane-enveloped state may help the virus to escape from the host immune system [25]. Balayan et al. [26] first isolated a novel viral pathogen, later identified as HEV, from the fecal extracts of patients with enterically transmitted non-A and non-B hepatitis (ET-NANBH). In 1983, immuno-electron microscopy (IEM) revealed non-enveloped viral particles with a density of 1.35 g/cm^3^ according to CsCl gradient centrifugation. In an in vitro infection model, if viral particles can be observed, it is considered direct evidence of infection. Hepatitis A virus (HAV) has been shown to have a capsular structure, but the hijacking of the cell membrane helps to evade neutralizing antibodies, thereby potentially enhancing viral transmission in the liver [27]. Many studies have shown that cell culture-derived HEV particles possess a lipid envelope. The density of HEV-infected A549 cells after centrifugation is 1.15–1.16 g/mL, which is lower than that of fecal particles. Studies have shown that the efficiency of HEV particles in serum bound to anti-ORF2 MAb and anti-ORF3 MAb is significantly improved after treatment with 5% Tween 20, with similar results to HEV particles produced in cell culture. The results suggest that HEV virions in serum have lipid-associated ORF3 proteins on their surfaces, similar to ORF3 proteins in the supernatant of cultured cells [28]. Another study [2] showed that infectious RNA from PLC/PRF/5 cell cultures was rich in the ORF3 protein and lipids, while the corresponding fraction from feces contained no lipids and a small amount of the ORF3 protein, indicating that its structure was different from that of the virions found in feces.

### 2.2. Genomic Structure and Viral Markers of HEV

**Figure 1:**
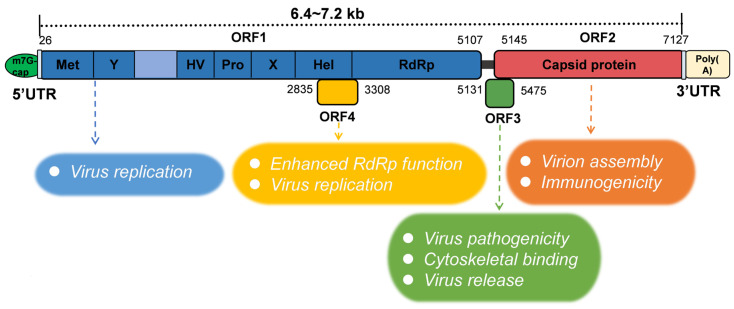
Schematic of HEV genomic organization and subgenomic RNA. ORF1 (nt 26-5107) is labeled above the genomic RNA box. Met, methyltransferase domain; Y, Y domain; HV, hypervariable region; Pro, proline-rich domain; X, X-domain; Hel, helicase; RdRp, RNA-dependent RNA polymerase. ORF2 (nt 5145-7127) and ORF3 (nt 5131-5475) are encoded by the same subgenomic RNA. The numbers above or below the RNA box indicate the number of nucleotides in the cDNA of HEV Sar55 (GenBank entry number AF444002). The HEV ORF function is shown in the figure below.

In the 1990s, Tam et al. [29] published the complete sequence of the HEV genome. Sequence analysis showed that HEV is a positive-stranded RNA (+ssRNA) virus with a full-length genome sequence of 6.4–7.2 kb [30,31], including three open reading frames (ORF1–3) and two short non-coding regions (NCRs) [32]. The latter encompasses an “m7G cap” structure in the 5′ NCR and a poly A tail at the 3′ end (Figure 1).

The ORF1 region is the longest among these three ORFs and is located at the 5′ end of the genome, encoding a non-structural protein of approximately 1693 amino acids [33]. The specific amino acid number of ORF1 was related to the strain. The computer-assisted assignment of ORF1 functional domains included methyltransferase (Met), Y domain (Y), papain-like cysteine protease (PCP), the hypervariable region (HV), macrodomain (X), helicase (Hel), and the RNA-dependent RNA polymerase (RdRp) [3,34]. However, recent studies have shown that the region within ORF1 that was previously described as a “PCP” domain does not encode proteases and therefore should no longer be referred to as a PCP domain [35]. A sequence analysis of several alphavirus-like superfamily viruses indicates that the Y domain is an extension of methyltransferase [36]. The function of ORF1 is thought to mainly support viral replication, including viral RNA capping and RNA unwinding [37,38]. ORF2 is located at the 3′ end of the HEV genome and encodes not only the viral capsid protein but also a glycosylated secretory protein responsible for virion assembly and immunogenicity [39,40,41]. The ORF2 structural protein is targeted by neutralizing antibodies and is also frequently used as an indicator of viral infection for in vitro infection models, as well as a reliable diagnostic marker [11]. The ORF2-specific monoclonal antibody can be used for the rapid immunofluorescence detection of an HEV antigen in the urine of HE patients. In addition, ORF2 is a candidate for vaccine production. In a recent study, the protein was expressed in *Nicotiana benthamiana* using *pEff*, a self-replicating vector of potato virus X. After purification, ORF2 produced 150–200 µg per 1 g of green leaf biomass, and immunized mice could induce high levels of HEV-specific serum antibodies [42]. The location and role of the ORF3 protein in viruses were primarily determined by infectious cloning. In 2000, Wang et al. [43] first completed the whole genome sequence of HEV-4, and found that the position of the ORF3 initiator of HEV-4 was different from that of the ORF3 of the HEV 1–3 genotypes reported previously. Several years later, American scholars confirmed this conclusion through experiments in Huh-7 cells [44]. The importance of ORF3 in HEV infection was verified in a macaque model, which also confirmed the uniqueness of the HEV-4 ORF3 initiation position. ORF2 and ORF3 proteins are translated from a single- and double-stranded subgenomic mRNA, and the methionine start codon of ORF3 is located 11 nucleotides upstream of ORF2 [31]. ORF3 encodes a protein of 112 to 114 amino acids depending on the HEV genotype. For example, the ORF3 of HEV-5 and HEV-6 encodes a protein of 112 amino acids. Although the exact function of the ORF3 protein is not clear, it was reported that it is related to the pathogenicity of the virus. In addition, it may be involved in binding to the cytoskeleton [45], possibly also contributing to the process of virus release [46,47]. Immunofluorescence results showed that the ORF3 protein accumulated in the cytoplasm of PLC/PRF/5 cells transfected with the ORF3 expression plasmid and PLC/PRF/5 cells inoculated with culture HEV, which supported the previous hypothesis that the ORF3 protein was related to the virus release from the infected cells [48]. Studies have proved that the palmitoylation of the two cysteine residues at the N-terminal of ORF3 is crucial for its secretion and the formation of quasi-enveloped viruses [49]. An unknown ORF of 158 amino acids was also predicted within ORF1, located in the +1 reading frame (bases 2835 to 3308 at the 5′ end, based on the ORF1 numbering) and designated ORF4. Some studies have found that ORF4 is specifically expressed by HEV-1 under cellular stress and has been shown to enhance the function of RdRp [50]. An ORF4 carrying the HEV genome will help to establish an efficient HEV-1 model. In addition, all other currently known proteins, or any yet to be identified, represent potential targets for pharmacological intervention strategies [51].

### 2.3. Hosts and Transmission Routes of HEV

The earliest technique for the isolation of HEV strains was proposed by Reyes et al. [52], who collected infected human stool samples from ET-NANBH outbreaks in different regions and used infectious bile to construct a recombinant complementary DNA library to identify similar sequences, after which the virus was officially named HEV. HEV is an emerging zoonotic pathogen with a wide host range, and various HEV strains can cross the species barrier. Since the first detection of HEV in domestic pigs from the United States in 1997 [53], it was shown that HEV can infect humans, pigs [12,54], rabbits [14], cattle, sheep, camels [15], monkeys [55], mice [42,56], ferrets [18], and deer [57]. Based on studies using the HEV-3 Kernow-C1 strain in a variety of cell lines [58], it was shown that HEV can attach to and enter human liver cancer cells (HepG2/C3A, Huh-7, PLC/PRF/5), human colorectal adenocarcinoma cells (Caco-2), human non-small-cell lung cancer cells (A549), deer liver cells, chicken liver cells, as well as monkey, cow, mouse, deer, chicken, cat, dog, and rabbit cells. This study laid a solid foundation for the development of cell culture models in vitro.

Studies indicate that there is only one serotype and eight genotypes of HEV, while all HEV isolates can infect mammals. At present, HEV has been isolated from various animals such as pigs [12], wild boars [13,59], rabbits [14], and camels [60]. The strains infecting humans mainly include HEV genotypes 1–4 [61], as well as the recently discovered HEV-7 [16]. At present, it is believed that HEV-1 and HEV-2 can only infect humans, and the infection rate of HEV-1 is higher than that of HEV-2 [50]. The majority of cases are in areas with poor medical and sanitary infrastructure, where the drinking water supply, coastal waters, and shellfish are often contaminated by human and animal feces, fomenting HEV transmission [62]. While HEV-3 and HEV-4 are zoonotic pathogens, the main risk factor is direct contact with or the consumption of pigs, wild boars [50], and deer [57], mostly constituting food-borne transmission, which is the main route of sporadic HE. The HEV-5 and HEV-6 strains have so far been detected only in wild boar populations in Japan [63]. In 2014, researchers first identified a novel HEV strain (designated HEV-7) in dromedary camels in Dubai [17]. Subsequent studies in 2016 detected another distinct genotype (HEV-8) in Bactrian camels in Xinjiang, China, further demonstrating the expanding diversity of HEV mammalian hosts [64]. Notably, a case study reported HEV-7 infection in a liver transplant recipient with frequent exposure to camel-derived products (meat and milk), providing the first evidence of zoonotic transmission potential for camelid HEV strains [16]. In addition, rat HEV was first identified in Germany in 2010 [65]. Rats can be infected with *Rocahepevirus ratti*. Recent studies have shown that rat HEV-C1 overflows and spreads from rodents to pigs, cats, and dogs [66,67].

HEV is the main cause of sporadic and epidemic hepatitis, and this disease is no longer limited to Asia and developing countries [62] but has become a concern in developed countries.

## 3. In Vitro Culture Models of HEV Infection

At present, many research teams have established in vitro cell culture systems and organoid models to isolate and culture HEV, which has greatly accelerated the research on the determinants and mechanisms of HEV infection in humans [31].

### 3.1. HEV Cell Culture Models

#### 3.1.1. Cell Culture Systems for Natural Virus Isolation

Cell culture is a central method in virological research [68], and since the discovery of HEV, researchers have attempted to propagate it in a variety of cell lines (Table 1). Primary cell culture models of non-human primates play an important role in understanding the replication of HEV and its pathogenic mechanism. The primary culture cell models of HEV that have been reported so far include green monkey kidney cells (BGMK), macaque kidney cells, and macaque hepatocytes, among which the primary macaque hepatocytes are the most widely used [69]. At present, the reported HEV passage cell culture models include PLC/PRF/5, Huh7, HepG2/C3A, HepaRG, Caco-2, A549, 2BS, FRhK-4, PICM-19, and BRS. Among them, the commonly used HEV passage cell lines are hepatocellular carcinoma cell lines and lung cancer cell lines.

HEV virions are stable in alkaline buffers and can maintain their integrity in the presence of magnesium or manganese ions [70], but its culture in vitro is difficult, and most HEV cell culture systems are limited by low viral titers and slow viral replication. The host cell type, medium composition and concentration, as well as the genetic modifications of the virus all affect the culture efficiency. In view of these challenges, it is of great significance to develop efficient HEV cell culture methods for studying its molecular biology and pathogenesis.

First, the selection of cell lines for HEV in vitro culture is critical. Capelli et al. [71,72] established a polarized hepatocyte model called HepG2/F2, which simulated the main physiological characteristics of hepatocyte growth in medium containing DMSO, after which they used this model to analyze the supernatants of the apical and basal sides of cells as well as the subcellular distribution of HEV. Another study established a PLC/PRF/5 cell culture model of HEV, which can scale up to 4 L and continuously produce HEV for 8 months [73]. This model was used to investigate HEV replication, infection, and antiviral mechanisms. However, PLC/PRF/5 cells produce HBs antigen. This could be a drawback for electron microscopy experiments. The HepG2/C3A cell line, a subclone derived from the hepatoma cell line HepG2 [74], can support the proliferation of HEV-3 Kernow C1/p6 relatively efficiently, but still cannot support the proliferation of HEV-1, and also cannot support virus proliferation following HEV-RNA transfection. Therefore, although there is no more general and efficient culture system based on the continuous subcloning of different HEV genotypes of liver cancer cells, the current system can also be used to study bottlenecks in host–HEV interactions. Moreover, different cell lines have different sensitivities to HEV. For example, the titer of focus-forming units (FFUs) formed by the Kernow-C1 strain in HepG2/C3A cells is about 7.5 times that formed in PLC/PRF/5, A549, and Caco-2 cells [58]. To explore the small animal model of HEV infection, Mongolian gerbils were inoculated intraperitoneally with HEV-5, HEV-7, HEV-8, rabbit HEV, or rat HEV [75]. Except for the rat HEV strain, the rest were all cultured in the human hepatoma cell line PLC/PRF/5 and inoculated with the cell culture supernatant. To generate a large number of V-105 strains for infection experiments, researchers intravenously inoculated Wistar rats with 10% tissue homogenate and allowed them to incubate for 30 days [75,76].

Secondly, the medium characteristics that affect the efficiency of HEV cultivation include the pH value, composition, and even different brands of the same medium. Some studies have increased the HEV TCID_50_ from 10 to 100 times by changing the composition of the medium, including fetal bovine serum (FBS) and dimethyl sulfoxide (DMSO) concentrations. It has been shown that decreasing the concentration of FBS, from 10% to 2%, reduces cell proliferation but maintains HEV replication in PLC/PRF/5 cells [77]. The results showed that HEV RNA production increased more than 10-fold after 1 or 6 h of seeding in WED (96% William’s medium E, 1% DMSO, 2% heat-inactivated exosome-free FBS, 1% PSA) cultured cells or 6 h of seeding in DSD (87% DMEM, 2% DMSO, 10% heat-inactivated FBS, 1% PSA) cultured cells compared with MCCI (2% FBS and 30 mM MgCl_2_). This result may be due to the fact that both WED and DSD contain DMSO, which promotes hepatocyte differentiation [72]. Cells isolated from human liver tumors (HepaRG cell line) express liver-specific functions and are sensitive to hepatitis B virus (HBV) infection [78]. Compared with the 100% enzyme-linked immunosorbent assay (ELISA) positive rate (EPR) of 1 × 10^6^ copies/mL of HEV inoculation, at a pH 3.0, a pH 11.0, at 56 °C, and under surfactant-free treatment conditions, the positivity rates of ELISA after 30 min of incubation were 100%, 75%, 37.5%, and 100% [73]. Compared with minimal essential medium (MEM), mixed DMEM/M199 increased the infection efficiency of HEV in PLC/PRF/5 cells [73]. Previous HEV isolation protocols typically used media supplemented with amphotericin B and 30 mM MgCl_2_ [70] or non-essential amino acids (NEAAs) [79], in addition to different antibiotics. Experiments have shown [80] that the addition of 10 mM CaCl_2_, KCl, K_2_SO_4_, MgCl_2_, MgSO_4,_ or Na_2_SO_4_ can increase the concentration of the HEV ORF2 antigen in the supernatant, while KH_2_PO_4_, NaCl, and Na_2_HPO_4_ can reduce the antigen concentration. Surprisingly, the addition of 2.5 mg/L of amphotericin B to the PLC/PRF/5 cell supernatant resulted in the greatest increase in HEV RNA and ORF2 antigen, while the combination of K_2_SO_4_ and CaCl_2_ had the most obvious effect on promoting HEV replication. Another study detected a more than 6-fold increase in HEV-3 replication in A549/D3 cells by adding three supplements, amphotericin B, MgCl_2,_ and DMSO, to the culture medium [81].

Thirdly, genetic modification efforts to date have generally failed to significantly improve the viral titer. The replication of the HEV genome requires the participation of ORF1 non-structural proteins, template RNA, and host factors [51]. A study [82] found that the R458K mutation in the Met region of HEV ORF1 can relieve the restriction of PRMT5/WDR77, so the R458K mutant has enhanced replication ability. Moreover, the Gluc activity of the Kernow C1/p6 Gluc R458K replicon mutant in WT cells or PRMT5 knockout cells was about two times higher than that of the Kernow C1/p6 Gluc WT replicon in WT cells. Given that rabbit HEV-3ra is evolutionarily closely related to human HEV-3, in order to determine the effect of HEV-3 RdRp mutations related to ribavirin (RBV) treatment failure on the replication of rabbit HEV-3ra [83], some scholars have established HEV-3ra infectious clones and successfully reproduced them in human liver cancer PLC/PRF/5 cells. A persistent viral infection was induced in inoculated rabbits, and it was found that the Y1320H/K1383N double mutant was significantly more sensitive to RBV treatment. These optimized methods can be applied to biosafety processes and the detection of novel antiviral drugs. Differentiated hepatoma cells (HepaRG) were infected with HEV-3 isolates from acute HE patients, and efficient replication was still maintained after seven consecutive passages [22].

#### 3.1.2. HEV Infectious Clonal Culture System

Although HEV patient isolates can be used for in vitro and in vivo infection studies, their genomes cannot be modified, limiting their use in studying the functions of viral proteins and non-coding regions. In order to overcome the challenge of viral propagation due to the low replication efficiency of clinical HEV isolates and their passaged progeny, infectious cDNA clones were developed. The construction process of viral infectious clones generally entails the cloning of the DNA or cDNA sequence of the virus into the relevant plasmid, after the genetic material of the virus is obtained through in vitro transcription or other methods to achieve virus rescue. In 1976, Goff et al. [84] pioneered the field of viral infectious cloning. As an alternative to conventional cell culture, infectious cDNA cloning was used to study viral thermostability, the factors affecting host range, the post-translational modifications of viral proteins, and the functions of different viral proteins.

HEV-1 only infects humans, but in vitro cell culture experiments showed that HEV-1 can also infect non-human cells. Emerson et al. [85] transfected 11 cell lines with HEV-1 infectious cDNA clones, including Huh-7, Caco-2, HepG2/C3A, PLC/PRF/5, A549, rat liver cancer cells (BRL3A), human liver cancer cells (Hepa 1–6), porcine kidney cells (PK), porcine testicular cells (ST), human skin fibroblasts (HS27), and African green monkey kidney cells (Vero). All the above cells expressed pORF2 and pORF3, but only PLC/PRF/5 and Huh-7 could produce an infectious progeny virus. However, the successful construction of the infectious cDNA clones of RNA viruses is a challenging process that is hampered by many obstacles, such as the instability of RNA viruses, unexpected mutations during RT-PCR and molecular cloning steps, the emergence of RNA mutations during in vitro transcription, as well as the specific “cap” and poly-A tail structures required for efficient replication. The 5′ cap structure is critical for the infectivity of hepatitis E virus (HEV). While uncapped HEV RNA retains some infectivity, its replication efficiency is significantly reduced compared to capped viral RNA [74]. In 2005, Huang et al. successfully constructed the first infectious clone of HEV-3 and demonstrated that pigs would develop viremia and other related symptoms after intrahepatic inoculation with “capped” HEV RNA. In some studies, Huh-7 was infected with the capped RNA transcript of the full-length cDNA clone, and the pig infectious cDNA clone pGEM4z-SAAS-JDY5 was successfully constructed [86]. Methods for the in vitro and in vivo rescue of infectious cDNA from the cloned HEV-4 TW6196E strain were subsequently developed [74], which confirmed that the cap-RNA transcript from the pHEV-4TW clone was replicable in Huh-7 and infectious in HepG2/C3A cells. Avian HEV (aHEV) belongs to the genus *Avihepevirus* of the family *Hepeviridae* and is found in chickens with hepatitis splenomegaly syndrome (HSS). Like porcine HEV derived from pigs, aHEV is genetically and antigenically related to human HEV. At least three aHEV in vitro culture systems have been successfully established, and full-length cDNA clones have been reported in the literature [87,88,89]. Studies have shown that the infectious clones of aHEV cDNA were constructed using viruses from the standard aHEV infectious stock [90], and the liver cells of Leghurian male hepatocellular carcinoma (LMH) chickens were transfected in vitro and directly inoculated into the livers of specific-pathogen-free (SPF) chickens [88]. Similarly, at least three other cell culture systems of rat HEV have been established, and cDNA clones have been constructed [47,91,92].

An ideal in vitro cell culture system for HEV should be able to support the simultaneous replication and infection processes of multiple HEV genotypes, thus achieving co-culture and providing a complete culture system for subsequent research on infectivity, disease diagnosis, and transmission. Okamoto et al. [70,93,94] obtained the full-length infectious cDNA clone of the HEV-3 isolate JE03-1760F and the HEV-4 strain from stool specimens, after which they successfully cultured the viruses in the human cell lines A549 and PLC/PRF/5. A group [95] isolated strain JE04-1601S from a patient infected with HEV-1. After an initial passage in PLC/PRF/5 and 12 successive passages in A549_1-1H8 cells, it was found that the replication efficiency of the JE04-1601S strain was significantly improved without cytopathic effects. This indicates that the virus adapted to grow in cell culture. Host-specific viral fragments and cytokines may affect the cross-species transmission of HEV. Using pSK-HEV2 as the backbone, 12 chimeric whole-genome clones of HEV-1/4 were constructed by replacing the structural regions (ORF2 and ORF3), non-structural region (ORF1), and non-coding regions (NCRs) with corresponding fragments of the HEV-4 clone. The resulting construct was then used to transfect human liver cancer cells (S10-3) and porcine kidney cells (PK-15) [96]. All chimeric vectors were found to replicate in S10-3, but only the two chimeric clones HEV-1 (HEV-4 5′ncr-orf1) and HEV-1 (HEV-4 ORF1) were found to replicate in PK-15 cells, which also demonstrated, for the first time, the critical role of the ORF1 polyprotein in crossing the species barrier in vitro.

**Table 1:** Cell culture models of different HEV genotypes.

HEV Genera	HEV Genotype	Strain	Year of Publication	Susceptible Cell Lines	cDNA Cloned or Not	Mode of Transformation	References
A	HEV-1	87A	1995	A549 *	No		[97]
A	HEV-1	87A	1999	2BS *	No		[98]
A	HEV-1	Sar55	2003	PLC/PRF/5 Huh-7	No		[86]
A	HEV-1	Sar55	2010	Caco-2 *	No		[99]
A	HEV-1	Sar55	2018	HLCs *	No		[100]
A	HEV-1	Sar55/S17	2016	M03.13 * BeWo * JEG-1 *	No	Introduced the S17 fragment from Kernow-C1/P6	[68,101]
A	HEV-1	JE04-1601S	2023	PLC/PRF/5 A549 *	pJE04-1601S_p12		[95]
A	HEV-2	MEX-14	2018	HLCs *	No		[102]
A	HEV-3	JE03-1760F	2007	PLC/PRF/5 A549 * HepG2/C3A PK15 * IBRS-2 * LLC-PK1 *	pJE03-1760F/wt		[93]
A	HEV-3	Kernow-C1	2011	HepG2/C3A (F2) A549 * Caco-2 * Huh-7 PLC/PRF/5	No	F2 subclone was isolated from the HepG2/C3A cell line and used in a polarized monolayer culture to achieve highly efficient HEV culture	[58,71]
A	HEV-3	Kernow-C1	2016	DBTRG * SK-N-MC * DAOY * U-373MG * M03.13 *	Kernow-C1-p6	All tested cell lines supported the replication of HEV RNA and demonstrated HEV entry into the oligodendrocyte line M03.13	[68]
A	HEV-3	Kernow-C1	2021	HepaRG	No	Remained infectious to pigs after 6 generations	[22]
A	HEV-3	SwJB-P5 SwJB-E10 SwJB-M8	2014	PHH	No		[102]
A	HEV-3	SAAS-JDY5	2014	Huh-7	pGEM4z-SAAS-JDY5		[87]
A	HEV-3ra	LR	2023	PLC/PRF/5	LR_Y1320H LR_K1383N LR_K1634G LR_K1634R		[84]
A	HEV-3ra	ME-2016-rab52	2022	HepG2	rab52LucA26 rab81LucA26		[103]
A	HEV-3ra	rbIM223LR	2021	PLC/PRF/5	pUC57-T7RHEV-LR		[104]
A	HEV-4	HE-JF5/15F	2009	PLC/PRF/5 A549 * HepG2/C3A PK15 * IBRS-2 *	HE-JF5/15F_p6		[94]
A	HEV-4	HB-3	2011	IBRS-2 * A549 *	No		[54]
A	HEV-4	TW6196E	2012	Huh-7 HepG2/C3A	pHEV-4TW		[74]
A	HEV-4	KM01	2024	HepG2	No		[20]
A	HEV-1 + HEV-4 chimeric virus	IND-SW-00-01	2016	S10-3 PK15 *	pSK-HEV2	All 12 chimeric vectors could replicate in S10-3 cells, but only 2 could replicate in PK15	[96]
A	HEV-5	JBOAR135-Shiz09	2018	PLC/PRF/5	G5 HEV		[75,105,106]
A	HEV-6	wbJHG_23	2024	PLC/PRF/5 A549 *	pwbJHG_23_P1 pwbJHG_23_P1-GAA		[59]
A	HEV-7	DcHEV-180c	2021	PLC/PRF/5	No		[75,106]
A	HEV-7	DcHEV-180c	2016	PLC/PRF/5	pUC57-T7DcHEV		[107]
A	HEV-8	M2	2021	PLC/PRF/5 A549 * Caco-2 *	pVL1393-G8n111ORF2		[75,106,108]
B	HEV_Avian	avian HEV-prototype	2005	LMH	pT7-aHEV-5 pTG-aHEV-10 pT7G-aHEV-6		[87]
B	HEV_Avian	avian HEV-VA	2011	LMH	pT7-aHEV-K		[89]
B	HEV_Avian	HH-F9	2015	LMH	pT11-aHEV-K		[88]
C	HEV-C1	R63	2015	PLC/PRF/5	pUC19-R63		[91]
C	HEV-C1	LA-B350	2016	Huh7 HepG2/C3A	pLA-B350 pLA-B350/luc		[92]
C	HEV-C1	ratELOMB-131	2018	PLC/PRF/5	pUC-ratELOMB-131L_wt		[47]

* indicates that the cell is not a human liver cancer cell line.

### 3.2. In Vitro Organoid HEV Infection Model

Although human liver cancer cell lines (such as PLC/PRF/5, Huh-7, HepaRG, HepG2, etc.) have been used to simulate hepatitis virus infection [58,85], these traditional two-dimensional cell culture systems still have certain limitations. For example, PLC/PRF/5 and A549 cells cannot simulate the infection of HEV in vivo under natural conditions, the RNA load of HEV produced in the supernatant of HepaRG cells is relatively low, etc. In addition, these systems lack the expression of crucial liver-specific genes and specific biological functions, which cannot reproduce the heterogeneity and complex structural characteristics of liver cells well. Recent breakthroughs in the research on liver and other tissue organoids have resulted in a new in vitro culture method for HEV, which is more economical and conforms to the requirements of animal welfare as an alternative to animal infection models.

#### 3.2.1. Current Status of HEV Organoid Infection Models

Organoids are in vitro 3D cell clusters differentiated from tissue-resident stem/progenitor cells, embryonic stem cells (ESCs), or induced pluripotent stem cells (iPSCs) with self-renewal and self-organization abilities [109]. Organoids can maintain their structure and function for extended periods of in vitro culture, whereby the interactions between cells recapitulate those of tissues and organs in vivo [110]. In recent years, the construction of the organoid models of the stomach [111], intestine [112], brain [113], and liver [114] has been reported in succession, which provides the possibility to study HEV infection in different organs.

An ideal organoid model should consist of more than one cell type with a self-renewal ability, combined with the ability to be induced to differentiate into other cell types. In a recent study, both undifferentiated cholangiocyte organoids and differentiated liver organoids were found to be susceptible to HEV-3 infection, which was consistent with the clinical observation of infected hepatic duct cells and hepatocytes in the liver of HE patients [23]. Subsequently, the team demonstrated the anti-HEV effect of ribavirin and mycophenolic acid (MPA) in organoids. This also greatly promoted the research on HEV host interaction and antiviral drug screening. Since HEV is mainly transmitted through the fecal–oral and urine–oral routes, the gut and liver are also barrier tissues. Moreover, as these tissues are anatomically and functionally linked, they are often referred to as the gut–liver axis. Once HEV in the gastrointestinal tract has gained access to the blood through the intestinal epithelium, it will reach the liver to infect hepatocytes, but the complexity of this axis cannot be reproduced by conventional cell culture systems [109], and the role of the gut in HEV-induced diseases remains unexplored. The complete replication cycle of HEV is supported by iPSC-derived hepatocyte-like cells (HLCs). Some studies established a novel liver organoid and intestinal culture system supporting authentic HEV infection by inducing human immortalized hepatocytes (C3A) to differentiate into HLCs and embedding them in Matrigel [109]. Another study established an HEV infection model of human intestinal organoids (HIEs) using differentiated 3D-HIEs [21]. Abundant HEV ORF2 antigen was detected in the infected HIEs, whereby HEV infection promoted the differentiation of cells into enteroendocrine cells, which supported infection by the virus. It is suggested that the use of intestinal organoids will contribute to a deeper understanding of HEV infection and disease.

#### 3.2.2. Novel Organoid Models for HEV Infection

Nusse et al. [115] described a long-term culture of primary mouse hepatocytes that retained many of their morphological and functional properties. Researchers constructed an HEV infection model in pregnant ICR mice and found that the abundance of LC3 protein in the placental tissue of mice was significantly reduced, indicating that placental tissue is also susceptible to HEV infection [56]. In addition to generating liver organoids from mouse and human cells, human bile duct organoids have also been established in rats [116], cats [117], and dogs [118]. Since several liver diseases progress in cats and dogs in a similar manner to humans, the use of organoids from these species may provide translatable therapeutic strategies for humans [119]. With the progress of 3D cell culture technology, an increasing number of organoid models have been established [115]. The development of mouse neural tube organoids [120,121] and organoid vascularization bioengineering technology [122] will promote and accelerate biomedical discovery and innovation. A recent study [123] found that HEV-3 can replicate in cultured human testicular tissue, primarily human testicular Sertoli cells, and the testicular tissues of immunosuppressed rabbit models. This resulted in a change in the inflammatory homeostasis of testicular Sertoli cells, providing an explanation for the clinical phenomenon of long-term semen contamination in chronic HEV infection [124]. The results suggest that close attention should be paid to the long-term reproductive system health status of male patients with chronic HEV infection and also provide a direction for the development of reproductive system organoids for the in vitro culture of HEV in the future.

To some extent, organoids can replace animal models in the study of viral infection and antiviral drug evaluation. Through the joint efforts of biologists and engineers, establishing and optimizing methods for more suitable organoids will help virologists study the mechanisms of HEV infection and pathogenesis, which will provide important clues for identifying new antiviral drug targets for HEV therapy.

## 4. Application of In Vitro Cell Culture Models of HEV Infection

**Figure 2:**
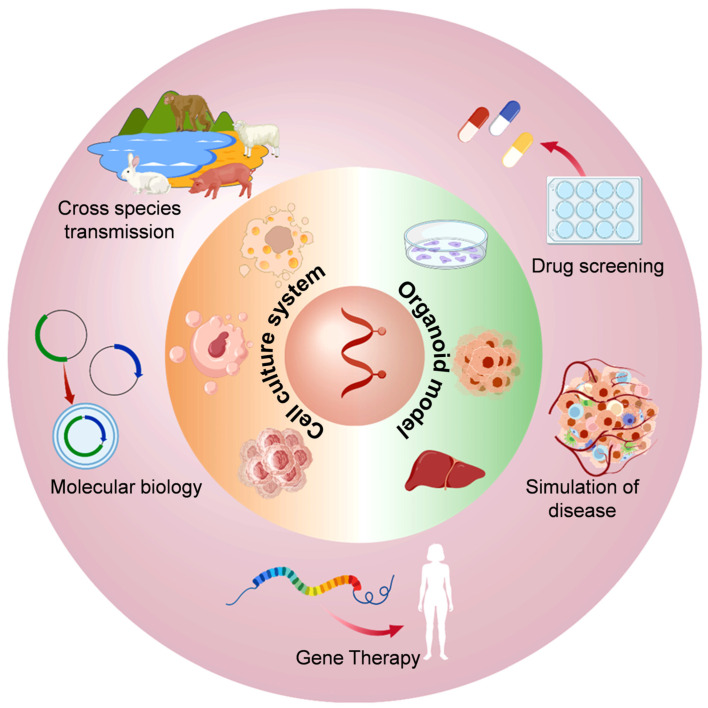
Application diagram of the in vitro cell culture model of HEV infection.

In recent years, multiple HEV culture models have been extensively studied from the aspects of molecular biology [74], disease simulation [21,85], pathogenesis [20,82], cross-species transmission [54,96,125], gene therapy [126], and drug screening [22,23] (Figure 2).

### 4.1. Molecular Biology Research on HEV

Cell culture models provide an important tool for studying the molecular biological characteristics of HEV. By using in vitro culture systems, researchers were able to analyze the HEV genome structure, replication mechanisms, transcription and translation processes, as well as the virus–host factor interactions in depth. In one study [20], researchers successfully infected HepG2 cells with HEV, and it was found that the degree of HEV infection was positively correlated with the expression of activated growth inhibitory family member 5 (ING5). Using PLC/PRF/5 and Huh7 cell lines, researchers revealed the function of the HEV ORF, with a key role in viral replication [43,70].

### 4.2. HEV Disease Simulation Studies

An HEV cell culture model was used to simulate the viral infection process to study the effects of the virus on host cells. For example, by using human liver cancer cell lines such as HepG2 and Huh7, as well as primary hepatocyte models, researchers were able to simulate the hepatocyte damage, inflammatory response, and immune response caused by HEV infection in vivo [58]. These models provide an important basis for understanding the pathophysiological mechanisms of HEV-associated hepatitis. More significantly, HEV tropism is not restricted to the human liver, as HEV can complete the entire viral life cycle in human oligodendrocytes (M03.13) [68], which supports HEV-associated neuropathological observations.

### 4.3. Research on the Cross-Species Transmission of HEV

HEV is a zoonotic virus, and cross-species transmission is an important epidemiological feature. By using appropriate cell culture models, researchers can assess the replication efficiency of different HEV genotypes in cells of different species, thereby revealing the molecular basis of cross-species transmission. HEV-3 remained infectious in pigs via oral inoculation after six passages in HepaRG cells [22]. In addition, HEV-4 (HB-3) was able to replicate in vitro in A549 and porcine kidney cells (IBRS-2) [54], suggesting that HEV can cross the species barrier and spread between pigs and humans. In order to determine the risk of cross-species transmission among rabbits, mice, and pigs, researchers inoculated pigs intravenously with American rabbit HEV, Chinese rabbit HEV, American rat HEV, or porcine HEV [125]. The results showed that only half of the pigs inoculated with rabbit HEV had low-level viremia and fecal virus shedding, indicating that HEV infection was active but not intense. The infection of pigs with rabbit HEV was further confirmed by the transmission of the virus found in pig feces to young rabbits, but no evidence of infection was found in pigs inoculated with rat HEV. In this study, the cross-species transmission ability of rabbit HEV was verified by fecal–oral transmission. Another study found that after inoculating Japanese white rabbits with HEV-5, HEV-7, and HEV-8, respectively, viral RNA was present in the fecal specimens of rabbits inoculated with HEV-8, and anti-HEV IgG antibodies were present in the serum. The results indicated that HEV-8 was a cross-species infection [106].

### 4.4. HEV Gene Therapy Research

HEV cell culture models provide a platform for the development of antiviral strategies based on gene therapy. For example, studies using CRISPR/Cas9 technology to target the HEV genome have been validated in an in vitro cell culture model. The results showed that the CRISPR/Cas9 system could efficiently target the HEV genome and introduce insertion or deletion mutations at specific positions, thereby inactivating the virus. Moreover, the effect was better than that of ribavirin treatment [100].

### 4.5. Anti-HEV Drug Screening

The cell culture models of HEV infection are an important tool for antiviral drug screening and evaluation. Using high-throughput screening techniques, researchers identified a variety of potential anti-HEV compounds, such as ribavirin, interferon, and novel small-molecule inhibitors. Researchers also investigated the antiviral effect of ribavirin in a model of persistent HEV infection established using the HepaRG cell line. Ribavirin was found to interfere with the activity of viral RNA-dependent RNA polymerase (RdRp) and possibly inhibit the translation process of viral proteins. As a result, it reduced the synthesis of viral structural proteins (such as ORF2 capsid protein), as well as non-structural proteins (such as ORF1 replicase), thereby inhibiting viral replication in cells [22].

## 5. Advantages, Disadvantages, and Prospects of In Vitro Culture Models of HEV Infection

Cell culture models can mimic HEV infection in vivo, but the difficulty of viral genome engineering has limited their application in the study of viral gene function. Nevertheless, the development of infectious clones can make up for the deficiency of in vitro cell culture systems in the field of hepatitis virus research. However, the construction of HEV infectious clones is still a great challenge due to the uncertain viral rescue efficiency and the need to introduce mutations to ensure the stable replication of a variety of viruses in the cell culture system. Three-dimensional culture in vitro can more closely mimic the environment of cells living in vivo, but liver organoids are still a long way from a fully mature artificial liver. Under normal physiological conditions, different types of hepatocytes work together to maintain the physiological and biochemical microenvironment of the liver, which supports cellular morphogenesis, migration, and the response to viral infection. Optimizing the construction of liver and other tissue organoids will promote the study of HEV infection and pathogenesis, as well as the identification and evaluation of antiviral drugs. Therefore, it is important to establish an efficient in vitro culture model for the study of HEV.

So far, a variety of in vitro infection models have played an important role. The improvement and optimization of HEV cell culture models, especially the development of liver and intestine organoids, provides a new method for HEV in vitro culture and reduces the need for experimental animals for research on HEV pathogenesis and drug screening, thereby promoting research on HE prevention and control.
